# ASO Author Reflections: First Do No Harm—Revisiting Adjuvant Hyperthermic Intraperitoneal Chemotherapy (HIPEC) Trials to Reduce the Risk of Peritoneal Metastasis

**DOI:** 10.1245/s10434-021-10263-z

**Published:** 2021-06-11

**Authors:** Can Yurttas, Alfred Königsrainer, Markus W. Löffler

**Affiliations:** 1 Department of General, Visceral and Transplant Surgery, University Hospital Tübingen, Tübingen, Germany; 2 German Cancer Consortium (DKTK) and German Cancer Research Center (DKFZ) Partner Site Tübingen, Tübingen, Germany; 3 Cluster of Excellence iFIT (EXC2180) “Image-Guided and Functionally Instructed Tumor Therapies”, University of Tübingen, Tübingen, Germany; 4 Interfaculty Institute for Cell Biology, Department of Immunology, University of Tübingen, Tübingen, Germany; 5 Department of Clinical Pharmacology, University Hospital Tübingen, Tübingen, Germany

## Past

Hyperthermic intraperitoneal chemotherapy (HIPEC) combined with cytoreductive surgery (CRS) was initially introduced as a multimodal treatment for pseudomyxoma peritonei and peritoneal mesothelioma. The approach did not only address a dire unmet medical need, because peritoneal metastases (PM) respond only poorly to systemic chemotherapy, but intra-abdominal chemotherapy application also convinces with a compelling theoretical rationale, namely direct high-dose drug exposure of remnant tumor and free cancer cells.

Due to the success of this treatment, compared with palliative chemotherapy as the standard of care and steadily growing evidence (e.g., reviewed for colorectal cancer (CRC) PM in Bijelic et al.^1^), including randomized controlled trials (RCT), the enthusiasm for this combined procedure has substantially increased and the indication spectrum has been extended. Against this background, HIPEC was not only suggested for selected patients in the therapeutic setting but also for tertiary prevention in cancer surgery, designed to prevent tumor dissemination and thereby aiming to reduce the risk of peritoneal recurrence after primary tumor resection. This also applies to pancreatic ductal adenocarcinoma (PDAC), which is a malignancy with a particularly poor prognosis, where gemcitabine HIPEC was recommended as a promising strategy and clinically used.^2^

## Present

Due to the lack of appropriate studies, evidence was unavailable that could have discerned between the contribution of CRS and a myriad of HIPEC procedures used over the years. Meanwhile a variety of well-designed RCTs has been published that do question the effectiveness of HIPEC, both in the therapeutic setting, as well as for tertiary prevention.^3^,^4^ As a result, it became evident that surgery is the mainstay of treatment and does improve patient survival, whereas HIPEC may not only prove futile but may also involve harm to patients.^3^ Admittedly, this conclusion so far only applies to short-term oxaliplatin HIPEC in CRC. Other studies can substantiate therapeutic benefits of HIPEC for highly selected patients with PM from ovarian cancer.^5^

However, such data still reproach us with the fact that HIPEC has been frequently applied without distinction. Thus, more than one blind spot prevails. For HIPEC convincing preclinical data and proof for clinical effectiveness are frequently elusive as well as adequate answers to the question which potential harm the treatment entails. The latter aspect seemed particularly relevant to us when intending to combine tertiary prevention with a high-risk surgery setting, as prevalent for instance in HIPEC after primary PDAC resection. Of note, here the risk-benefit ratio is also altered compared with HIPEC used for therapeutic purposes.

Therefore, a prospective, open-label, phase I/II trial has been designed to assess the 30-day mortality rate, treatment feasibility, and the adverse events profile of complete macroscopic resection of PDAC followed by one-hour gemcitabine HIPEC. As a result, no adverse event above grade 3 related to HIPEC occurred during the trial and no patient died within 1-month after surgery. Accordingly, this trial attests to the safety of HIPEC application after PDAC resection, which is reassuring.^6^

## Future

The current status quo has demonstrated that a compelling rationale is not sufficient but robust data are required before the broad implementation of interventions.^3^ Although surgical and pharmacological treatments are very different, taking respective therapeutic effects and harms apart from each other remains challenging. This should certainly not be considered as an excuse to omit future studies enabling the illumination of our blind spots. In HIPEC, including treatments for tertiary prevention, it seems advisable to take a step backwards and to carefully reassess its principles and fundamentals. Furthermore, additional innovation and usage of the ample novel treatment approaches meanwhile available (e.g., immunotherapies or precision medicine) might outperform traditional cytostatic drugs—the same applies to novel clinical trial designs.^7^ We ultimately believe that HIPEC, particularly when tertiary prevention is concerned, should be prospectively investigated for feasibility and safety before other clinical endpoints are addressed. In this regard, this present trial might even be regarded as an example for future treatment attempts.^6^

## References

[1] Bijelic L, Ramos I, Goere D (2021). The Landmark Series: surgical treatment of colorectal cancer peritoneal metastases. Ann Surg Oncol..

[2] Tentes AA, Stamou K, Pallas N, Karamveri C, Kyziridis D, Hristakis C (2016). The effect of hyperthermic intraoperative intraperitoneal chemotherapy (HIPEC) as an adjuvant in patients with resectable pancreatic cancer. Int J Hyperthermia..

[3] Quenet F, Elias D, Roca L (2021). Cytoreductive surgery plus hyperthermic intraperitoneal chemotherapy versus cytoreductive surgery alone for colorectal peritoneal metastases (PRODIGE 7): a multicentre, randomised, open-label, phase 3 trial. Lancet Oncol..

[4] Klaver CEL, Wisselink DD, Punt CJA (2019). Adjuvant hyperthermic intraperitoneal chemotherapy in patients with locally advanced colon cancer (COLOPEC): a multicentre, open-label, randomised trial. Lancet Gastroenterol Hepatol..

[5] van Driel WJ, Koole SN, Sikorska K (2018). Hyperthermic Intraperitoneal Chemotherapy in Ovarian Cancer. N Engl J Med..

[6] Yurttas C, Horvath P, Fischer I (2021). A prospective phase I/II open label pilot trial to assess the safety of hyperthermic intraperitoneal chemotherapy after oncological resection of pancreatic adenocarcinoma. Ann Surg Oncol..

[7] Yurttas C, Fisher OM, Cortés-Guiral D (2020). Cytoreductive surgery and HIPEC in colorectal cancer—did we get hold of the wrong end of the stick. Memo—Magazine of European Medical Oncology.

